# The complete mitochondrial genome of the common sea slater, *Ligia oceanica *(Crustacea, Isopoda) bears a novel gene order and unusual control region features

**DOI:** 10.1186/1471-2164-7-241

**Published:** 2006-09-20

**Authors:** Fabian Kilpert, Lars Podsiadlowski

**Affiliations:** 1 Department of Animal Systematics and Evolution, Institute of Biology, Freie Universität Berlin, Konigin-Luise-Str. 1-3, D-14195 Berlin, Germany

## Abstract

**Background:**

Sequence data and other characters from mitochondrial genomes (gene translocations, secondary structure of RNA molecules) are useful in phylogenetic studies among metazoan animals from population to phylum level. Moreover, the comparison of complete mitochondrial sequences gives valuable information about the evolution of small genomes, e.g. about different mechanisms of gene translocation, gene duplication and gene loss, or concerning nucleotide frequency biases.

The Peracarida (gammarids, isopods, etc.) comprise about 21,000 species of crustaceans, living in many environments from deep sea floor to arid terrestrial habitats. *Ligia oceanica *is a terrestrial isopod living at rocky seashores of the european North Sea and Atlantic coastlines.

**Results:**

The study reveals the first complete mitochondrial DNA sequence from a peracarid crustacean. The mitochondrial genome of *Ligia oceanica *is a circular double-stranded DNA molecule, with a size of 15,289 bp. It shows several changes in mitochondrial gene order compared to other crustacean species. An overview about mitochondrial gene order of all crustacean taxa yet sequenced is also presented. The largest non-coding part (the putative mitochondrial control region) of the mitochondrial genome of *Ligia oceanica *is unexpectedly not AT-rich compared to the remainder of the genome. It bears two repeat regions (4× 10 bp and 3× 64 bp), and a GC-rich hairpin-like secondary structure. Some of the transfer RNAs show secondary structures which derive from the usual cloverleaf pattern. While some tRNA genes are putative targets for RNA editing, *trnR *could not be localized at all.

**Conclusion:**

Gene order is not conserved among Peracarida, not even among isopods. The two isopod species *Ligia oceanica *and *Idotea baltica *show a similarly derived gene order, compared to the arthropod ground pattern and to the amphipod *Parhyale hawaiiensis*, suggesting that most of the translocation events were already present the last common ancestor of these isopods. Beyond that, the positions of three tRNA genes differ in the two isopod species. Strand bias in nucleotide frequency is reversed in both isopod species compared to other Malacostraca. This is probably due to a reversal of the replication origin, which is further supported by the fact that the hairpin structure typically found in the control region shows a reversed orientation in the isopod species, compared to other crustaceans.

## Background

The metazoan mitochondrial genome is a circular double stranded DNA molecule of about 12–20 kb length. Due to the important role of mitochondria for cell metabolism its gene content is highly conserved and typically contains the same 37 genes: 13 protein-coding genes, two ribosomal genes and 22 transfer RNA genes [1]. In addition one A+T-rich non-coding part is present which contains essential regulatory elements for transcription and replication. It is therefore referred to as the mitochondrial control region [1]. The organization of the mtDNA is compact with very little non-coding sequences between genes, even gene overlaps by a few nucleotides are commonly found (especially at the boundaries between *nad4/nad4L *and *atp6/atp8*). As a result the gene order of mitochondrial genomes is relatively stable because rearrangements are likely to disrupt genes. Thus changes in gene order are relatively rare, whereas tRNA genes more frequently change their position than larger protein-coding and rRNA genes [2]. Mollusca [3-6], Brachiopoda [7-10] and Nematoda [11-14] represent phyla where a lot of rearrangements of mitochondrial genomes were reported, whereas in Chordata only few changes in gene order were found [2]. Among arthropods a lot of species have retained the arthropod ground pattern (or a slight modification in Hexapods and Crustacea), while some taxa show frequent genome rearrangements, e.g. Myriapoda [15-17], Hymenoptera [18,19], Acari [20,21], and Araneae [22,23]. Peracarid crustaceans seem to represent another example, as two partially sequenced mitochondrial genomes [24,25] exhibit strong differences between each other and from the arthropod ground pattern.

Mitochondrial genomes offer a broad range of characters to study phylogenetic relationships of animal taxa. Besides nucleotide and amino acid sequences, tRNA secondary structures [26], deviations from the universal genetic code [27,28], as well as changes in the mitochondrial gene order [29,30] are successfully used as characters in phylogenetic inference. Especially the changes in gene order prove as extremely reliable phylogenetic characters because the probability that homoplastic translocations occur in closely related taxa is very low. Dowton et al. [31] calculated a chance of 1/2664 for a single gene translocation event occuring independently in two mitochondrial genomes (starting from the same gene order in both). However, this probability could be underestimated according to yet unidentified constraints on modes of gene rearrangements and should be handled with care. With about 21,000 known species peracarids comprise approximately one third of all crustacean species so far described. Within Crustacea the isopods form the largest subtaxon (10,000 species). Isopods show an amazing ecological diversity and morphological flexibility. They are common around the globe, their habitats range from deep sea plains over freshwater wells to terrestrial, even arid deserts. Although to date 30 complete mitochondrial genomes from crustaceans are available – thereof 16 from malacostracan species – a complete mitochondrial sequence of a peracarid species is still missing. Recent sequencing efforts with the amphipod *Parhyale hawaiiensis *[24] and the isopod *Idotea baltica *[25] produced almost complete genome records, lacking only the control region and some of the tRNA genes. Here we present the first complete sequence of a peracarid mitochondrial genome. *Ligia oceanica *(Isopoda: Oniscidea) is a terrestrial species living on rocky seashore habitats. It is found from Norway to Iceland in the north, around the british islands and the north sea coasts south to northern Spain and Portugal. We discuss changes in gene order compared to other crustacean taxa and give an overview about genome rearrangements in Crustacea. In addition we compare nucleotide composition of isopod mitochondrial genes and tRNA secondary structure, and describe in detail uncommon features of the mitochondrial control region from *Ligia oceanica*.

## Results and discussion

### Genome organization

**Figure 1:**
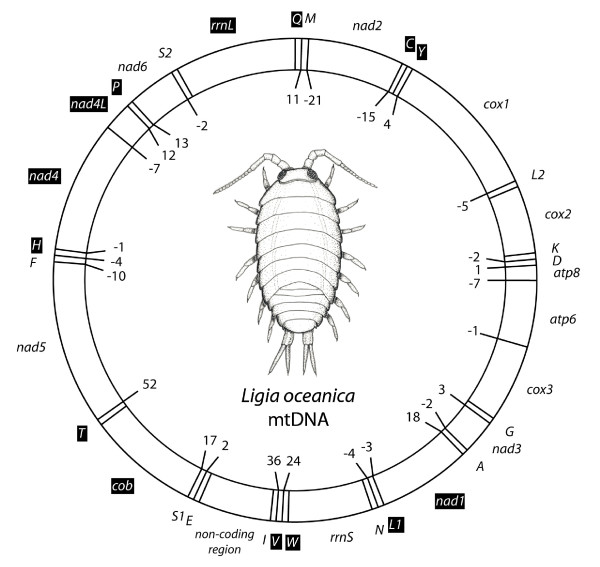
Map of the mitochondrial genome of *Ligia oceanica*. Transfer-RNAs are represented by their one-letter amino acid code. Inverted (white on black) legends indicate genes located on (-)strand. Numbers specify the length of noncoding sequences or the extent of gene overlaps (negative values), respectively.

The complete mitochondrial genome sequence of *L. oceanica *has an overall length of 15,289 bp [GenBank:DQ442914]. Successfully accomplished PCRs have proven a circular organization of the molecule (Fig. 1). Although this is the general state of metazoan mitochondrial DNA, it is mentioned here, because there is evidence for a linear organization in a related species, *Armadillidum vulgare *(Isopoda: Oniscidea) [32]. All 13 protein coding subunits which are usually found in metazoan mitochondrial genomes are present, as well the two rRNA subunits (Table 1). In contrast only 21 tRNA genes instead of the typical number of 22 were identified (see below). In addition one major non-coding sequence was detected, which presumably contains the origin of replication and regulatory elements for transcription (mitochondrial control region). There are small gene overlaps at 14 gene borders. The largest has a length of 15 nucleotides (between *nad2 *and *trnC*). Some small non-coding sequences exist which occur quite often in arthropod mitochondrial genomes. The largest extends up to 52 nucleotides and is located between *trnT *and *nad5*.

### Protein-coding genes

The A+T content of the protein coding genes of the *L. oceanica *mitochondrial genome is with 60.1% (A = 28.6; C = 16.7%; G = 23.2%; T = 31.5%) at the lower end observed for malacostracan species. The values range from a 60.0% minimum given by *Cherax destructor *[33] to a 69.3% maximum by *Penaeus monodon *[34]. Whereas the majority of the 13 protein coding genes show usual start codons for mtDNA, two genes begin with exceptional codons (Table 1). The gene *atp8 *probably starts with codon GTG. Although there is an ATA codon nine bp downstream from this start codon, alignments with *atp8 *amino acid sequences from other arthropods suggest the presence of more amino acids in the starting region. GTG is probably also in use as start codon in mitochondrial genes from *Idotea baltica *(*nad1 *and *cox2*) [25]. The second gene with an apparently exceptional codon is *cox1*, which starts with ACG. Although this seems to be unusual in metazoan mitochondrial genomes, almost all other malacostracan crustaceans studied so far have this start codon for *cox1*. The only known exception concerns the crab *Portunus trituberculatus *[35]. Two of the protein coding genes show truncated stop codons. The gene for *nad1 *terminates with TA whereas *cox3 *bears a single thymine at its end. This is a well known phenomenon in the mitochondrial genome and is frequently reported for several species. The stop codons are very likely completed by post-transcriptional polyadenylation, so that each transcript finally obtains a functional UAA terminal codon [36].

In most arthropods there is a strand specific bias in nucleotide frequencies [37,38]. In detail the (+)strand contains more cytosine and adenine, while the (-)strand consequently is more rich in guanine and thymine. Some taxa show a reversal in that strand bias, among them the isopod *Idotea baltica *[25]. Strand bias is best reflected in GC skew [37,39] of mitochondrial genes (Table 2). In *Ligia oceanica*, as well as in *Idotea baltica*, GC skew is positive in (+)strand encoded genes, while it is negative in (-)strand encoded genes. This is in contrast to most other malacostracan crustaceans and is probably due to an inversion of the mitochondrial control region, or at least the replication origin [37]. Further evidence comes from sequence analysis of the control region (see below).

**Figure 2:**
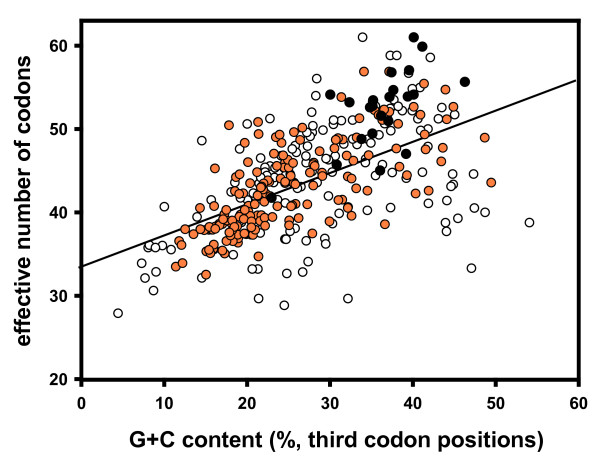
Effective number of codons versus G+C content in third codon position in crustacean mitochondrial genes. All species with complete mitochondrial genome entries are included (for a species list, GenBank accession numbers and single values see supplementary Table 1). For each species eleven mitochondrial protein-coding genes were evaluated and plotted (all except *nad4L *and *atp8*, which contain less than 100 codons). Black dots: genes from Isopoda; orange dots: genes from all other Malacostraca; white dots: genes from Crustacea excl. Malacostraca. Regression line with r^2 ^= 0.3381; p < 0.01.

The effective number of codons (ENC) is a statistic describing how far codon usage in protein-coding genes departs from the equal usage of all synonymous codons [40]. Its range lies between 20 (when only one codon is used for each amino acid) and 62 (when all synonymous codons are equally in use). The latter departs from the usual value of 61 for nuclear genes as in invertebrate mitochondrial genomes 62 codons are in use (instead being a stop codon, UGA codes for tryptophane in the invertebrate mitochondrial code). The ENC of all published crustacean mitochondrial genomes was determined for all genes (Fig. 2), except *nad4L *and *atp8*, because these genes are too short (less than 100 codons) to get proper results. A positive correlation with G+C content in third codon positions was revealed (r2 = 0.3381; p < 0.01). There is no obvious difference seen between malacostracan and other crustaceans. Genes from the two isopod species (*Ligia oceanica *and *Idotea baltica*) are of higher G+C content and therefore show a higher than average number of effective codons. For numbers of effective codons for individual species and genes, as well as GenBank accession numbers see additional file 1.

### Transfer RNAs

We identified 21 out of normally found 22 transfer-RNA genes in the mitochondrial genome of *Ligia oceanica*. Despite extensive efforts to find secondary structures in non-coding regions the gene *trnR *was not found in the mitochondrial genomes sequence. By all means tRNA-Arg is essential for maintaining translation of mitochondrial gene products, so it has to be either imported into the mitochondrion, or its gene exists in the mitochondrial genome, but is subject to extensive RNA editing and therefore not identifiable by now.

**Figure 3:**
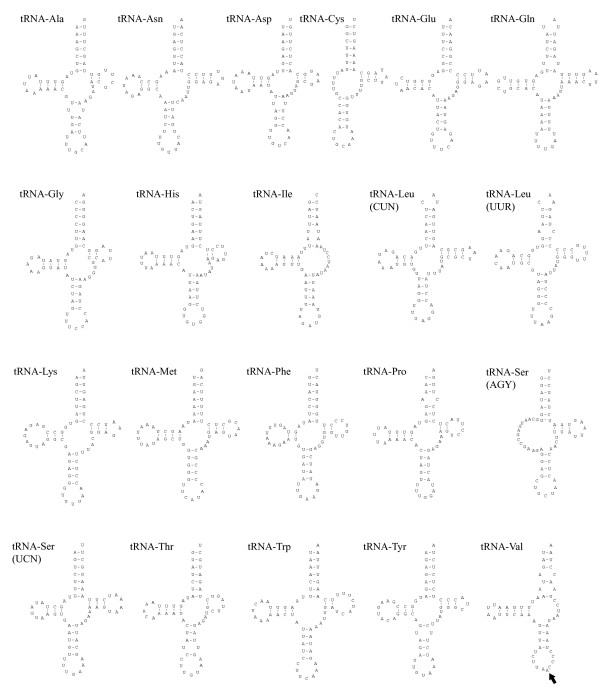
Plots of the mitochondrial tRNAs found in *Ligia oceanica*. An additional nucleotide, probably deleted by RNA editing, is found in the anticodon loop of *trnV *(arrow).

Transfer-RNA genes are spread over the entire genome and are located on both strands (Fig. 1, Table 1). 14 of them were identified using tRNAscan-SE 1.21 [41]. The other seven tRNA genes (*trnD, trnC, trnE*, *trnI*, *trnF*, *trnS1*, *trnW*) were found by eye inspection of otherwise non-coding regions. Some of the putative secondary structures derive from the usual cloverleaf pattern (Fig. 3): tRNA-Cys and tRNA-Ser(AGY) lack the DHU-arm. The loss of this arm in tRNA-Ser(AGY) was also observed in many other arthropod species, among malacostracan crustaceans *Pseudocarcinas gigas *and *Macrobrachium rosenbergi *[42], *Euphausia superba *[43], *Cherax destructor *[33], *Penaeus monodon *[34], and *Portunus trituberculatus *[35]. In contrast to that, the derived structure of tRNA-Cys seems to be unique among malacostracan species studied so far. Transfer-RNA-Val and tRNA-Ile miss the TΨC-arm. Again these features are not seen in other malacostracan crustaceans.

A misplaced adenine was recorded in the anticodon loop of tRNA-Val. Its existence has been proven by repeated sequencing of different PCR-products. To assure the functionality of this gene a correctional RNA editing must be presumed in which a single nucleotide is removed. Similar post-transcriptional events with insertion and deletion of single nucleotides are known from the mitochondrial mRNAs of trypanosomes [44], tRNA editing was demonstrated in the centipede *Lithobius forficatus *[15]. In addition several mismatches are found in tRNA stems, most of them in the acceptor stem (Fig. 3: tRNA-Gln, tRNA-Ile, tRNA-Leu1, tRNA-Leu2, tRNA-Pro, tRNA-Val, and in the anticodon stem (Fig. 3: tRNA-Ala, tRNA-Asp, tRNA-Thr, tRNA-Tyr). Such mismatches were also reported from other animal mitochondrial tRNAs and are probably further subjects to RNA editing [15,22,45].

### Control region and repetitive sequences

**Figure 4:**
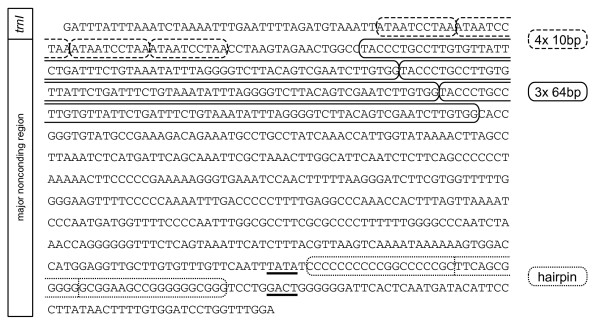
Sequence of *trnI *and the major non-coding region (control region) of *Ligia oceanica*. The region contains two sections with tandem repeats (4× 10 bp, 3× 64 bp) and a GC-rich region containing the putative hairpin structure (see also Fig. 5).

There is one major non-coding region of 737 bp length located between *trnI *and *trnE*. It is assumed to be the mitochondrial control region. At its boundary to *trnI *it contains two sections with repetitive sequences (Fig. 4). The first consists of a series of four completely matching sequences of 10 bp each and extends into *trnI*. The second section is formed by a consecutive triplicate 64 bp segment. No similarities of these sequences to any other mitochondrial gene could be identified. Only a few other mitochondrial genomes were shown to contain any repeat region: rabbit mitochondrial genomes show repeated 153 bp motifs in their mitochondrial control region, varying in copy number between different individuals or tissues [46]; the highly aberrant mitochondrial genome of the brachiopod *Lingula anatina *posesses ten different unassigned repeated elements ranging in size between 28 bp and 1092 bp and in copy number between 2 and 11 [7]. Also some insects show tandem repetitions in mitochondrial DNA [47].

**Figure 5:**
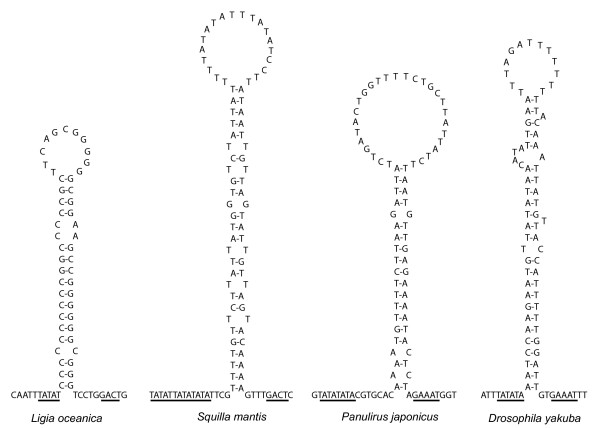
Hairpin structures in the mitochondrial control regions of *Ligia oceanica *(Isopoda), *Squilla mantis *(Stomatopoda), *Panulirus japonicus *(Decapoda) and *Drosophila yakuba *(Hexapoda). Conserved motifs in 5'- and 3'-flanking sequences are underlined. *Drosophila yakuba *structure according to [76], the other structures were deduced from GenBank entries.

Contrary to expectations the A+T content in the control region (55.8%) is lower than in other parts of the genome (protein coding genes: 60.1%). In contrast most other arthropods have an A+T-rich control region. While the repeat region is A+T-rich (70.3%), a 65 bp region near the 3'-end of the control region has an A+T content of only 14.1% (Fig. 4). That region is putatively folded into a hairpin-like structure with a stem consisting of 19 paired nucleotides (two mismatches) and a loop consisting of 11 nucleotides (Fig. 5). This hairpin-like structure highly resembles stem-loop structures known from insect mitochondrial control regions which have stems ranging between 15–30 bp and loops of about 9–15 nucleotides [48]. Similar stem-loop structures were found in other crustacean species, like the mantis shrimp *Squilla mantis *and the spiny lobster *Panulirus japonicus *(Fig 5). The flanking sequences around the stem region show conserved motifs: 5'-flanking sequences show a TATA element, while 3'-flanking sequence contains a GACT in *Ligia *and *Squilla*, while the GAAAT motif typical for insects is found in *Panulirus*. It is assumed that these structures are of functional importance in conjunction with the origin of replication [48]. In *Ligia *the flanking motifs are found in opposite direction and strand compared to that of *Squilla *and *Panulirus*. This fact gives direct evidence for an inversion of the control region in isopods (in addition to the reversed strand bias of nucleotide frequency mentioned above).

### An overview about gene translocations in Crustacea

**Figure 6:**
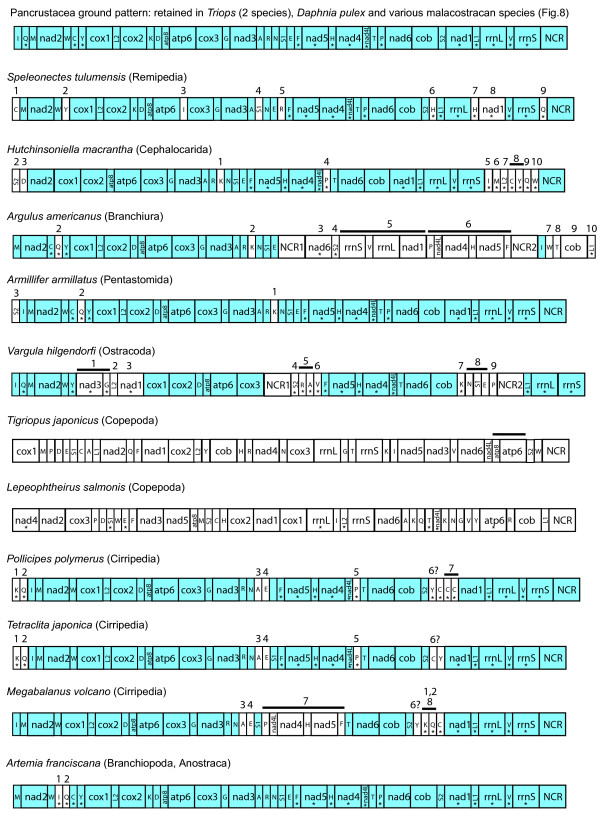
Gene order of mitochondrial genomes from Crustacea (excl. Malacostraca). All species with complete mitochondrial genomes are listed. White colours indicate changes compared to the pancrustacean ground pattern. Numbers refer to different gene translocation events (compare Fig. 7), horizontal lines combine adjacent genes, which were probably subject to a joint translocation. NCR = major non-coding region, the putative mitochondrial control region. Asterisks indicate genes located on (-)-strand. For GenBank accession numbers see supplementary Table 1.

Crustacean systematics is far from being settled. While Malacostraca seems to be a well defined clade, the interrelationships between crustacean subtaxa is under debate and even monophyly of Crustacea is doubtful, with respect to the position of Hexapoda, which are probably the next relatives to a crustacean subtaxon (Malacostraca, Branchiopoda or Copepoda) [24,34,49-51]. Mitochondrial genome rearrangements may serve as phylogenetic markers which support sistergroup relationships among Crustacea. From 35 species of Crustcea complete or almost complete mitochondrial genome sequences are recorded in GenBank. Gene order is not conserved among these taxa: only 13 species show no changes compared to the pancrustacean ground pattern (Fig. 6 and 8: Pancrustacea ground pattern). Transfer-RNA genes are more often translocated than other genes, probably because of their small size.

Crustacea and Hexapoda (united as Pancrustacea [30,52,53] or Tetraconata [54]) share the same ground pattern in mitochondrial gene order [30]. It differs from the euarthropod ground pattern [55] by the position of one tRNA gene: *trnL2 *is located between *cox1 *and *cox2*, whereas in Chelicerata, Myriapoda and Onychophora *trnL2 *is located between *nad1 *and *rrnl*, adjacent to *trnL1 *[29,30]. Among other data, mitochondrial gene translocations have shown that the enigmatic Remipedia and Pentastomida definitely belong to Pancrustacea, as they show the above mentioned translocation of *trnL2 *[56]. Only three crustacean species do not show this character: the cephalocarid *Hutchinsoniella macrantha *[56], where *trnL2 *probably is secondarily translocated to another position (Fig. 6, No. 7 from *Hutchinsoniella*), and the two copepod species *Tigriopus japonicus *[57] and *Lepeophtheirus salmonis *[58], which underwent a complete shuffling of the mitochondrial genome.

**Figure 7:**
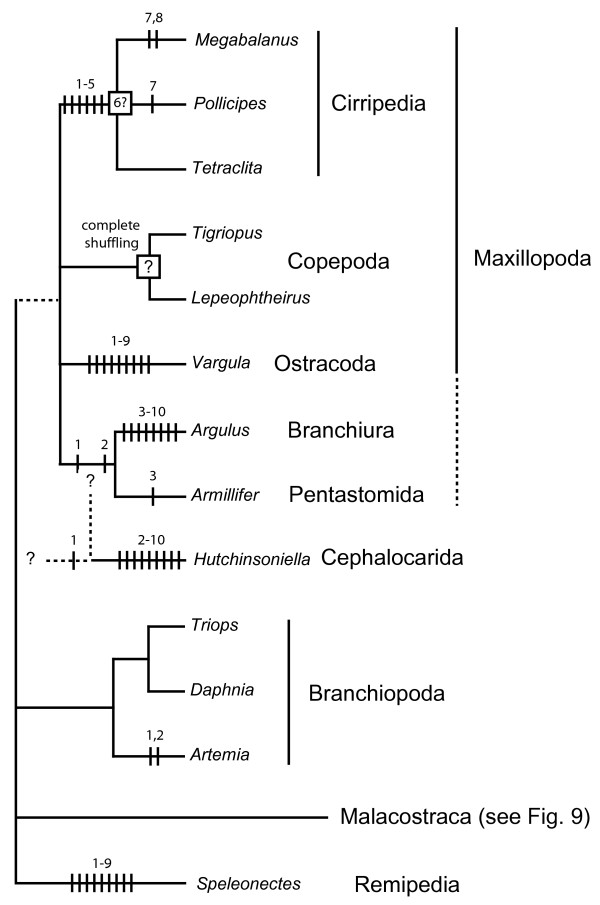
Phylogenetic tree of Crustacea. Only those species with GenBank entries for complete mitochondrial genomes are included. Hypotheses of gene translocation events are mapped to the corresponding clades (numbers correspond to those in Fig. 6). Due to the uncertain homology, translocations of the major non-coding region were not considered.

Three species (belonging to Cephalocarida, Branchiura and Pentatomida) share a translocation of *trnK *to a position between *trnR *and *trnN *[56]. Among these, the tongue worm *Armillifer armillatus *and the fish louse *Argulus americanus *share one further translocation (*trnQ*), together with mtDNA sequence analysis supporting a close relationship between Pentastomida and Branchiura [51,56]. That was already discussed according to sperm morphology [59,60] and 18S molecular sequence data [61]. *trnK *is also translocated in all other taxa refered to as members of "Maxillopoda", a systematic unit only weakly based on morphological characters. However, Ostracoda, Copepoda and Cirripedia each show different positions for *trnK *compared to the above mentioned taxa, so there is no good reason to take this as a homology. Because of contrary results from morphological and sequence based analyses [51,62] it is also questionable to unite the Cephalocarida with Branchiura and Pentastomida to one clade, solely based on the common translocation of *trnK *(No. 1 for *Hutchinsoniella */*Argulus*/*Armillifer *in Fig. 6 and Fig. 7).

The three species of Cirripedia [56], [GenBank:NC_006293; GenBank:NC_008974] share several translocations of tRNA genes (*trnA*, *trnE*, *trnP*). Another series of events is difficult to reconstruct: *trnC *and *trnY *are translocated in all three species to a position between *trnS2 *and *nad1*, but in different order and on different strands (referred to as No. 6 from Cirripedia in Fig. 6 and 7). In addition one species shows a triplication of *trnC *(No. 7 from *Pollicipes polymerus *in Fig. 6 and 7). Two further differences are reported in *Megabalanus volcano*: an inversion of a block of five genes (No. 7) and the probable translocation of *trnK *and *trnQ *to a position between *trnY *and *trnC *(No. 8 in Fig. 6 and 7). An alternative explanation is that *trnK/trnQ *were primarily translocated to that position seen in *Megabalanus *and secondarily translocated to the position seen in *Pollicipes *and *Tetraclita*. With data from these three species alone, it is not possible to reconstruct a ground pattern of mitochondrial gene order of Cirripedia with respect to the position of *trnK*, *trnQ*, *trnY *and *trnC*.

A lot of further translocation events are recorded only in single species, making them useless in phylogenetic analysis of the actual data set. Large genome rearrangements involving also protein-coding genes are seen in the branchiuran *Argulus americanus *[56], and in the ostracod *Vargula hilgendorfi *[63], both accompanied by a duplication of the control region. But the highest degree of genome rearrangement was found in the two copepod species [57,58], where a complete reshuffling of the mitochondrial genomes has led to a gene order with almost no similarities between the two species and to other crustaceans. Partial genomes from two other copepod species revealed even more rearrangements [64]. In contrast three species of Branchiopoda have retained the pancrustacean ground pattern. The fourth species, *Artemia franciscana *shows two tRNA gene translocations (*trnI, trnQ*) [65].

**Figure 8:**
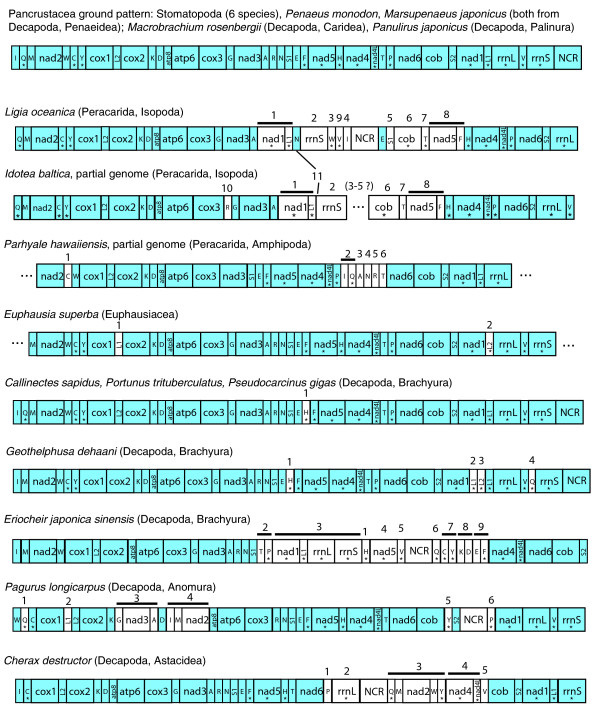
Gene order of mitochondrial genomes from malacostracan crustaceans. All species with complete or almost complete mitochondrial genomes are listed. White colours indicate changes compared to the pancrustacean ground pattern. Numbers refer to different gene translocation events (compare Fig. 9), horizontal lines combine adjacent genes, which were probably subject to a joint translocation. NCR = major non-coding region, the putative mitochondrial control region. Asterisks indicate genes located on (-)-strand. *Idotea baltica *[GenBank:DQ442915]; *Parhyale hawaiiensis *[GenBank:AY639937]; *Euphausia superba *[GenBank: AB084378]; for all other GenBank accession numbers see supplementary Table 1.

**Figure 9:**
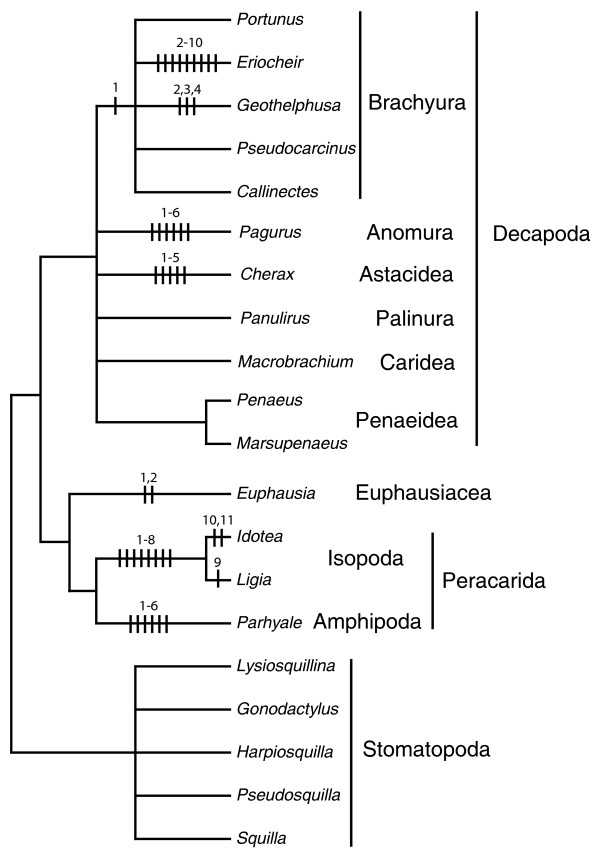
Phylogenetic tree of Malacostraca. Only those species with GenBank entries for complete or almost complete mitochondrial genomes are included. Hypotheses of gene translocation events are mapped to the corresponding clades (numbers correspond to those in Fig. 8). Due to the uncertain homology, translocations of the major non-coding region were not considered. Phylogeny according to [77].

Among Malacostraca 10 from 19 species have retained the pancrustacean ground pattern: six mantis shrimps (Stomatopoda) and four members of Decapoda (Fig. 8 and 9). Among Decapoda independent translocation events changed gene order in *Pagurus longicarpus *[66], *Cherax destructor *[33], and Brachyura. The four species of Brachyura share a translocation of *trnH*, the freshwater crabs *Geothelphusa dehaani *[67] and *Eriocheir japonica *[68] show further translocations. In the Euphausiacea *Euphausia superba *a swap between *trnL1 *and *trnL2 *seems to have happened, probably preceded by a gene duplication [43].

Besides the two isopod species (*Ligia oceanica*, *Idotea baltica*), only one other peracarid mitochondrial sequence, from the amphipod *Parhyale hawaiiensis*, was published before [24]. From six gene translocations that must be assumed to get the gene order of *Parhyale hawaiiensis*, none is shared with the mitochondrial genomes of isopods (Fig. 8 and 9). Only *trnI *is translocated in both taxa, but as its new position is different in *Parhyale hawaiiensis *and *Ligia oceanica *(and not known in *Idotea baltica*), there is no reason to presume that a translocation had already happened in their common ancestor. Instead we assume an independent translocation of this gene in isopods and amphipods. This implies, that the ground pattern of gene order in Peracarida must be identical to that of Pancrustacea.

### Gene translocations in isopods

A comparison of the complete mitochondrial genome of *L. oceanica *with the ancestral state of pancrustacea [30] demonstrates several changes in gene order (Fig. 8). All in all 11 genes (*cob*, *nad1*, *nad5*, *rrnS*, *trnI*, *trnL1*, *trnF*, *trnS1*, *trnT*, *trnW*, *trnV*) and the control region are found in other relative positions than reported in other malacostracan crustaceans. By reason of parsimony we assume that these positional changes were due to nine gene translocations (Fig. 8, 9; No. 1–9) and a translocation of the major non-coding region (NCR). The genes *nad5 *and *trnF*, as well as *nad1 *and *trnL1 *retained their adjacent positions, so that they probably were translocated as a block. The other genes were most likely repositioned by single translocation events. Five translocations led also to inversion of genes to the complementary strand: *cob*, *trnT *and *trnW *changed from the (+)strand to the (-)strand, whereas *rrnS *and the block of *nad*5 and *trnF *were inverted from (-)strand to (+)strand.

From the 11 genes being translocated in *Ligia oceanica*, seven are also found in the same new positions in the mitochondrial genome of *Idotea baltica *(Fig. 8) [25]. In addition the mitochondrial control region, *trnW*, *trnI *and *trnS1 *are not found in their original position in *Idotea baltica*, but will probably be found in the region not sequenced yet – between *rrnS *and *cob*, similar to *Ligia oceanica*. Of all genes translocated in *Ligia oceanica *only *trnV *is found in its original position in *Idotea baltica*. So of nine gene translocation events supposed for *Ligia oceanica*, eight must already have happended in the common ancestor of both species (Fig. 8, 9; No. 1–8). The derived gene order is probably the ground pattern for an isopod subtaxon comprised of Oniscidea and Valvifera (and probably more taxa). Translocation of *trnV *probably happened after the split of the oniscidean and valviferan lineages (Fig. 9, No. 9). In contrast, translocation of *trnN *probably happened in the lineage leading to *Idotea baltica *(Fig. 9, No.11). It is located in original position in *Ligia oceanica*, but missing in that position in *Idotea baltica*. We do not know about the fate of *trnR *in *Ligia oceanica*, but in *Idotea baltica *it was also subject to a translocation (Fig. 9, No. 10).

It is noticeable that in *Ligia oceanica *all translocated genes found their new position in a segment comprising about one third of the complete genome (between *trnA *and *trnH*). This area bears a cluster of tRNA genes in the ancestral gene order of arthropods. It seems to be a "hot spot" of genome rearrangements in arthropods [19,20].

## Conclusion

The first complete mitochondrial genome sequence of a peracarid arthropod, the isopod *Ligia oceanica*, shows the usual compact and circular organization known from other Metazoa. Gene order is not conserved among peracarids and even not among isopods. In *Ligia oceanica *11 genes plus the control region have changed their relative positions in comparison to the pancrustacean ground pattern, implying to be the result of nine gene translocation events. No gene translocation is shared with the amphipod *Parhyale hawaiiensis*, whereas eight gene translocations were probably already present in the common ancestor of *Ligia oceanica *and another isopod, *Idotea baltica*. Both isopod mitochondrial genomes differ by the position of three tRNA genes (*trnR*, *trnV*, *trnN*) and share an inverted strand bias of nucleotide frequencies compared to other malacostracan crustaceans. Reason for this is probably an inversion of the replication origin. This is confirmed by the fact that the typical hairpin-like secondary structure commonly found in mitochondrial control regions is found in opposite orientation compared to other crustacean species.

A broad survey of mitochondrial gene rearrangements in Crustacea reveals a great variation of gene order. Characters derived from gene order (= gene translocations, inversions or duplications) do not solve overall phylogenetic relationships between major crustacean subtaxa. However, they will probably be helpful in analyses of internal phylogeny of some of these subtaxa (Cirripedia, Brachyura, Peracarida), when more data will be provided.

## Methods

### Sample and DNA extraction

A specimen of *Ligia oceanica *originally collected at the coast of the North Sea island Helgoland (Germany) and preserved in 99% ethanol was utilized for the DNA extraction process. 2–3 pleopods were applied to the DNeasy Tissue Kit (Qiagen, Hilden, Germany) following the manufacturer's protocol to receive the total genomic DNA.

### PCR primers

The first partial mitogenomic sequences were obtained by using two insect based primer pairs N4 + 16S2 and CytB + N4(87) [69]. Additional intragenetic parts of *cox1*, *cox3*, *nd4*, *nad5*, *rrnL *and *rrnS *were determined by using six crustacean primer pairs [25,70]. The remaining gaps were closed by making use of specific primer pairs designed according to the sequences of the aforementioned genes (for primer sequences see additional file 2). Larger PCR products were sequenced by primer walking strategy. To abbreviate this longsome process two longer PCR products were sequenced with the primer pairs S1–S19 from a set of primers which was successfully applied to decapod crustaceans [71]. All primers were purchased from Metabion (München, Germany).

### PCR and purification of PCR products

The PCRs were performed with an Eppendorf Mastercycler or Eppendorf Mastercycler Gradient. The cycling was set up with an initial denaturation step at 94°C for 2 minutes, followed by 40 cycles comprising denaturation at 94°C for 30 seconds, annealing at 45–52°C (primer specific) for 1 minute and elongation at 72° for 2–5 minutes depending on the expected length of the PCR product. The process was completed with a final elongation at 68°C for 2 minutes. The reaction volume amounted 50 μl containing 1 μl dNTP mix (Eppendorf), 0.25 μl HotMaster*Taq *DNA polymerase (5 U/μl; Eppendorf), 5 μl HotMaster*Taq *buffer (Eppendorf), 1 μl primer mix (10 μM each), 1 μl DNA template and 41.75 μl sterilized distilled water (Eppendorf). The PCR products were separated with a 1% TBE agarose gel, stained with ethidium bromide and inspected subsequently under UV transillumination.

For the purification of the PCR Products the QIAquick PCR Purification Kit (Qiagen) as well as the Blue Matrix DNA Purification Kit (Eurx) were used. Abiding to the manufacturers protocols both kits produced equivalent yields. All PCR products were stored at -20°C until sequencing was performed.

### Cloning and transformation

In one case a single PCR fragment, due to low sequence signal quality, ranging from *rrnS *to *cob*, which contained the major non-coding region, was sequenced after cloning with the pGEM-T Easy Vector System (Promega). We followed the manufacturers protocol with the exception that half volumes (5 μl) were used for the cloning reaction. For the transformation *Escherichia coli *XL-gold (Stratagene) were used. Colonies that contained recombinant plasmids were identified with selection plates (LB/ampicillin/IPTG/X-Gal). To verify insertion of the PCR product, few cells were applied to a Colony PCR using the vector primers M13F and M13R. The reaction volume amounted 20 μl with aforementioned PCR ingredients added proportionally. The colony-cycling consisted of an initial denaturation step (10 minutes, 95°C) followed by 25 cycles of denaturation (30 seconds, 94°C), annealing (30 seconds, 46°C) and elongation (4 minutes, 68°C). The colony PCR was closed by a final elongation (3 minutes, 68°C). The result was inspected with an agarose gel under UV transillumination. Positive tested colonies were proliferated in a LB/ampicillin Medium. Subsequently the plasmids were extracted with the Quantum Prep Kit (Bio Rad) and finally stored at -20°C.

### Sequencing and sequence analysis

Cycle sequencing reactions were performed with the CEQ DTCS Quick Start Kit (Beckman Coulter) following the manufacturers protocols. The same primers and thermocyclers were used as in PCRs. The temperature profile included 30 cycles comprising denaturation at 94°C for 20 seconds, annealing at 45–52°C (primer specific) for 20 seconds and elongation at 60°C for 4 minutes. Plasmids were preheated additionally before the sequencing reaction (96°C for 1 minute). The separation was executed by a CEQ 8000 cappillary sequencer (Beckman Coulter) and analyzed with the appendant CEQ software (software version: 5.0.360, instrument version: 6.0.2).

### Gene annotation and sequence analysis

The alignment of the fragments to complete the whole mitochondrial DNA sequence was done in BioEdit 7.0.5.2 [72]. Each partial sequence was ascertained twice at least to prevent sequencing faults. Ambiguous base pairs were validated manually referring to chromatograms. Gene identification was determined by BLAST search on GenBank databases [73] and by comparison to the mitochondrial Genome of *Drosophila yakuba *(NC001322). Boundaries of the protein coding genes were determined with a multiple alignment of other crustacean amino acid sequences. It was assumed that they were specified by the first start and stop codons in frame. Transfer RNA genes were determined with tRNAscan-SE 1.21 [41] or by eye inspection for anti-codon sequences and secondary structures in regions between identified genes. Hairpin structures in non-coding regions were also identified by eye inspection. The control region and RNA genes were assumed to extend to adjacent genes, due to the lack of resources for a better determination of their boundaries. Nucleotide frequencies of protein coding and RNA genes were calculated with the DAMBE software package [74], effective number of codons was determined according to [40] with INCA 1.20 [75]. The complete genome sequence is submitted to NCBI GenBank [GenBank:DQ442914].

## Abbreviations

A, adenine; *atp6 *and *8*, genes encoding ATPase subunit 6 and 8; bp, base pairs; *cox1-3*, genes encoding cytochrome oxidase subnunits I-III; *cob*, gene encoding cytochrome b; C, cytosine; ENC, effective number of codons; G, guanine; mtDNA, mitochondrial DNA; *nad1-6 *and *nad4L*, genes encoding NADH dehydrogenase subunits 1–6 and 4L; nt, nucleotide(s); NCR, non-coding region; PCR, polymerase chain reaction; rRNA, ribosomal RNA; *rrnl*, large rRNA subunit (gene); *rrnS*, small rRNA subunit (gene); T, thymine; tRNA, transfer RNA; *trnX *(where X is replaced by one letter amino acid code), tRNA gene.

## Authors' contributions

FK did the majority of the laboratory work and the primary sequence analysis, LP was the initiator and supervisor of this work. Final analyses of data, discussion of results and drawing of the manuscript was done by both authors in equal shares.

## Supplementary Material

Additional File 1 Effective numbers of codons used in mitochondrial protein-coding genes of various crustacean taxa. These numbers are the data base for figure 2. Click here for file

Additional File 2 PCR primers used to amplify mitochondrial gene fragments from *Ligia oceanica*. Click here for file

## Figures and Tables

**Table 1:** Gene content of the mitochondrial genome of *Ligia oceanica*.

Gene	Strand	GenBank position no.	Size (nts)	Size (aa)	Start codon	Stop codon	Intergenic nucleotides
*trnQ*	-	1–63	63				11
*trnM*	+	75–140	66				-21
*nad2*	+	120–1142	1023	340	ATG	TAA	-15
*trnC*	-	1128–1181	54				0
*trnY*	-	1182–1243	62				4
*cox1*	+	1248–2786	1539	512	ACG	TAA	-5
*trnL2-UUR*	+	2782–2843	62				0
*cox2*	+	2844–3527	684	227	ATA	TAG	0
*trnK*	+	3528–3590	63				-2
*trnD*	+	3589–3648	60				1
*atp8*	+	3650–3805	156	51	GTG	TAA	-7
*atp6*	+	3799–4470	672	223	ATG	TAA	-1
*cox3*	+	4470–5256	787	262	ATG	T	0
*trnG*	+	5257–5317	61				3
*nad3*	+	5321–5671	351	116	ATT	TAA	-2
*trnA*	+	5670–5729	60				18
*nad1*	-	5748–6691	944	314	ATA	TA	-3
*trnL1-CUN*	-	6689–6750	62				-4
*trnN*	+	6747–6810	64				*
*rrnS*	+	6811–7660	850				*
*trnW*	-	7661–7726	66				24
*trnV*	-	7751–7806	56				36
*trnI*	+	7843–7899	57				*
non-coding region		7900–8636	737				*
*trnE*	+	8637–8696	60				2
*trnS1-AGY*	+	8699–8760	62				17
*cob*	-	8778–9911	1134	377	ATA	TAA	0
*trnT*	-	9912–9971	60				52
*nad5*	+	10024–11688	1665	554	ATT	TAG	-10
*trnF*	+	11679–11741	63				-4
*trnH*	-	11738–11800	63				-1
*nad4*	-	11800–13134	1335	444	ATG	TAA	-7
*nad4L*	-	13128–13418	291	96	ATA	TAA	12
*trnP*	-	13431–13492	62				13
*nad6*	+	13506–13994	489	162	ATT	TAG	-2
*trnS2-UCN*	+	13993–14055	63				*
*rrnL*	-	14056–15289	1234				*

* Gene borders defined by borders to adjacent genes.

**Table 2:** Nucleotide frequencies, AT content, AT- and GC-skew for mitochondrial genes of *L. oceanica*.

**Gene (strand)**	**Nucleotide frequency**				**%AT**	**AT skew**	**GC skew**	**GC skew**
	**A**	**C**	**G**	**T**			*L. oceanica*	*I. baltica*
*atp6 *(+)	0.244	0.205	0.222	0.329	57.3	-0.148	0.038	0,012
*atp8 *(+)	0.288	0.157	0.157	0.399	68.6	-0.162	0.000	0,100
*cox1 *(+)	0.236	0.190	0.224	0.350	58.6	-0.195	0.083	0,122
*cox2 *(+)	0.253	0.183	0.228	0.336	58.9	-0.142	0.111	0,169
*cox3 *(+)	0.219	0.212	0.234	0.336	55.4	-0.211	0.048	0,029
*cob *(-)	0.250	0.248	0.153	0.348	59.9	-0.164	**-0.235**	**-0,212**
*nad1 *(-)	0.236	0.205	0.213	0.346	58.3	-0.188	**0.020**	**-0,090**
*nad2 *(+)	0.269	0.149	0.236	0.347	61.6	-0.127	0.226	0,299
*nad3 *(+)	0.219	0.194	0.268	0.319	53.9	-0.185	0.161	0,320
*nad4 *(-)	0.273	0.229	0.142	0.357	63.0	-0.134	**-0.235**	**-0,247**
*nad4L *(-)	0.261	0.196	0.155	0.388	65.0	-0.196	**-0.118**	**-0,204**
*nad5 *(+)	0.275	0.127	0.255	0.343	61.7	-0.111	0.335	0,246
*nad6 *(+)	0.268	0.125	0.233	0.374	64.2	-0.166	0.303	0,240
*rrnL *(-)	0.345	0.186	0.163	0.306	65.2	0.060	**-0.065**	**-0,103**
*rrnS *(+)	0.324	0.187	0.212	0.278	60.1	0.076	0.062	0,098
CR	0.240	0.231	0.212	0.318	55.8	-0.139	-0.043	n.d.
total (+)	0.292	0.169	0.222	0.317	60.9	-0.041	0.136	n.d.

GC-skew from *Idotea baltica *genes for comparison. GC-skews from genes coding on (-)strand are shown in bold numbers.
