# Supplementary material for: The complete mitochondrial genome of the common sea slater, Ligia oceanica (Crustacea, Isopoda) bears a novel gene order and unusual control region features
Source: BMC Genomics. 2006 Sep 20;7:241. doi: 10.1186/1471-2164-7-241 (PMC1590035; doi:10.1186/1471-2164-7-241)
Supplement: Additional File 1 — Effective numbers of codons used in mitochondrial protein-coding genes of various crustacean taxa. These numbers are the data base for figure 2. [file 1471-2164-7-241-S1.pdf]

Additional file 1:

Number of effective codons used in mitochondrial protein-coding genes of Crustacea.

Species	Taxon	Acc.Number	*atp6*	*cox1*	*cox2*	*cox3*	*cob*	*nad1*	*nad2*	*nad3*	*nad4*	*nad5*	*nad6*
Speleonectes tulumensis	Remipedia	NC_005938	41,8	32,8	29,7	42,4	36,9	36,2	34,1	36,6	38,1	41,1	36,8
Hutchinsoniella macr.	Cephalocarida	NC_005937	32,7	34,9	33,2	34,3	37,9	41,5	28,8	32,1	45,6	45,4	39,5
Armillifer armillatus	Pentastomida	NC_005934	39,3	41,4	44,6	35,2	38,8	33,3	43,4	40,0	37,7	38,8	43,1
Argulus americanus	Branchiura	NC_005935	33,9	32,9	35,8	33,6	36,0	36,1	30,6	27,9	35,5	32,1	40,7
Vargula hilgendorfii	Ostracoda	NC_005306	50,6	49,8	51,2	49,9	47,9	44,1	44,8	40,5	41,1	44,8	49,8
Lepeophtheirus salmonis	Copepoda	NC_007215	43,6	50,6	51,1	45,6	48,1	46,4	52,7	39,9	46,0	46,3	37,7
Tigriopus japonicus	Copepoda	NC_003979	56,7	51,3	52,2	55,8	55,0	52,6	51,9	40,6	51,7	54,8	58,6
Megabalanus volcano	Cirripedia	NC_006293	44,2	42,5	47,6	41,7	48,0	37,1	44,4	37,9	43,5	44,8	49,1
Pollicipes polymerus	Cirripedia	NC_005936	50,4	44,6	49,6	50,1	48,2	44,9	46,0	43,1	48,8	42,0	52,0
Tetraclita japonica	Cirripedia	NC_008974	43,6	46,5	44,4	50,2	45,7	38,7	49,2	49,3	44,8	42,9	56,0
Artemia franciscana	Branchiopoda	NC_001620	48,0	47,2	46,8	54,4	48,8	52,2	54,0	61,0	53,3	49,9	53,8
Daphnia pulex	Branchiopoda	NC_000844	52,7	53,5	58,8	53,7	48,3	46,4	50,0	51,0	52,5	52,1	40,2
Triops cancriformis	Branchiopoda	NC_004465	46,8	41,7	45,3	49,2	42,9	38,4	41,2	48,6	42,3	38,8	51,2
Triops longicaudatus	Branchiopoda	NC_006079	41,7	38,9	43,6	40,8	44,2	37,5	46,5	50,6	41,5	41,5	36,8
Gonodactylus chiragra	Stomatopoda	NC_007442	49,0	43,5	45,4	40,8	44,8	40,6	42,9	49,6	39,0	42,0	47,3
Harpiosquilla harpax	Stomatopoda	NC_006916	39,8	40,3	40,1	39,5	47,1	44,4	37,3	43,5	39,7	38,6	32,5
Lysiosquillina maculata	Stomatopoda	NC_007443	46,8	50,6	44,1	51,1	47,7	49,4	46,1	45,8	52,3	48,8	51,7
Squilla empusa	Stomatopoda	NC_007444	46,8	43,7	37,5	41,2	48,7	43,4	41,0	34,7	39,5	38,5	44,6
Squilla mantis	Stomatopoda	NC_006081	42,0	43,0	40,3	42,0	50,2	37,6	37,9	45,3	38,4	38,2	50,9
Callinectes sapidus	Decapoda	NC_006281	43,7	41,0	46,0	46,9	39,1	40,8	43,5	43,2	36,4	41,3	40,9
Cherax destructor	Decapoda	NC_011243	55,5	51,7	52,7	51,0	54,7	44,3	49,0	47,8	52,1	51,1	43,6
Eriocheir sinensis	Decapoda	NC_006992	42,3	40,8	48,3	43,7	43,9	36,9	37,4	49,4	43,5	37,3	35,6
Geothelphusa dehaani	Decapoda	NC_007379	40,5	39,6	37,9	40,7	38,3	38,6	38,8	36,6	35,6	37,4	33,5
Macrobrachium ros.	Decapoda	NC_006880	47,6	42,3	42,6	45,1	38,6	39,6	44,5	51,2	40,5	41,5	41,5
Marsupenaeus japonicus	Decapoda	NC_007010	46,3	42,8	45,6	45,6	48,9	48,7	45,9	44,4	40,7	44,8	39,7
Pagurus longicarpus	Decapoda	NC_003058	40,8	37,9	38,0	34,2	38,3	37,4	38,3	50,5	35,1	35,3	39,5
Panulirus japonicus	Decapoda	NC_004251	49,3	47,7	56,9	56,9	46,9	52,3	51,3	53,8	49,0	46,2	45,5
Penaeus monodon	Decapoda	NC_002184	35,1	42,6	36,6	38,0	42,3	36,1	39,0	38,8	35,4	33,9	36,1
Portunus trituberculatus	Decapoda	NC_005037	42,2	39,6	39,0	40,8	38,5	37,3	39,4	44,8	37,2	38,0	35,5
Pseudocarcinus gigas	Decapoda	NC_006891	46,0	39,2	38,9	46,7	41,0	39,2	43,1	44,2	37,9	38,3	39,3
Idotea baltica	Peracarida	DQ442915	53,2	52,6	45,0	53,9	51,0	55,7	45,7	61,0	52,8	49,5	41,8
Ligia italica	Peracarida	DQ442914	56,8	53,5	54,7	59,9	51,6	57,1	53,9	54,1	54,1	48,8	47,0
