# Supplementary material for: The complete mitochondrial genome of the common sea slater, Ligia oceanica (Crustacea, Isopoda) bears a novel gene order and unusual control region features
Source: BMC Genomics. 2006 Sep 20;7:241. doi: 10.1186/1471-2164-7-241 (PMC1590035; doi:10.1186/1471-2164-7-241)
Supplement: Additional File 2 — PCR primers used to amplify mitochondrial gene fragments from Ligia oceanica. [file 1471-2164-7-241-S2.pdf]

Additional file 2:

PCR primers used to amplify mitochondrial gene fragments from *Ligia oceanica*

Primer	*Nucleotide sequence (5'-3')*	*Reference*
CB2H	TCCTCAAAATGATATTTGTCCTCA	[69]
N4(87)	TCAGCTAATATAGCAGCTCC	[69]
16S2	GCGACCTCGATGTTGGATTAA	[69]
N4	GGAGCTTCAACATGAGCTTT	[69]
crust-12f	CAGCAKYCGCGGTTAKAC	[70]
crust-12sr	ACACCTACTWTGTTACGACTTATCTC	[70]
crust-16sf	TGACYGTGCDAAGGTAGC	this study
crust-16sr	CCGGTCTGAACTCAYATC	[70]
crust-cox1f	ACTAATCACAARGAYATTGG	[70]
crust-cox1r	TAGTCTGAGTANCGTCGWGG	[70]
crust-cox3f	ATAATTCAATGATGACGAGA	[70]
crust-cox3r	CCAATAATWACATGWAGACC	[70]
crust-nd4f	TTGAGGTTAYCAGCCYG	[70]
crust-nd4r	ATATGAGCYACAGAAGARTAAGC	[70]
crust-nd5f	AGAATTCTACTAGGDTGRGATGG	[70]
crust-nd5r	AAAGAGCCTTAAATAAAGCATG	[70]
Lo-12s-f	AGGAGCAGGTGGGTTACAATC	this study
Lo-12sf-r	CTTTGGGTTTGAAGTACATAGC	this study
Lo-12sf-2	TATCTTTGAAGGATAATAGTTTTTAG	this study
Lo-12sf-3	AAATGCCTGCCTATCAAACC	this study
Lo-12sfr-2	TTACCTCAACTTGACAGATAAATGTG	this study
Lo-12sfr-3	CCTTGCTGGGTAGATTACGGTC	this study
Lo-16s-r	ATCTTAAAGGCTTACGAAATTCAG	this study
Lo-C1f-2	TTTCTTTGCATTTAGCTGGTG	this study
Lo-C1f-r	GGGGAAAGGCTATATCAGGAG	this study
Lo-C1r-2	AACCCCAATACCCCAATG	this study
Lo-C3-12	CTACTGGGAGTGTATTTTAGTCGTC	this study
Lo-C3f_r	CTACAGGGAGGTCAGATTCTAC	this study
Lo-C3f-2	GGAGTGTATTTTAGTCGTCTCTTC	this study
Lo-Cb-f	GTCCTACCACTACTCTCACACCTG	this study
Lo-Cbf-2	CCCGAATGATACTATCTATTTGCC	this study
Lo-Cbf-3	ACAGTAATGTGTTAGACACCTCCG	this study
Lo-CB-r	CAGGATATTTTTCTTGACTTTTAGG	this study
Lo-Co3-c	ATTATCTTTTTGGTATTTGATGTGG	this study
Lo-control-12s	CTTTAAAGGTTCTAAGGGTATAAGG	this study
Lo-control-cyb	AATTGGAGCCCGACCC	this study
Lo-CyB-N5	CCAGATCGACCCCAACG	this study
Lo-H4251	CAAAAAAAGGAAAAGAAGAATAGGAC	this study
Lo-L3400	TGTGGGAGAGGTTTTCAGCC	this study
Lo-L3401	ATTTGTTTGTTCTCGCCCAG	this study
Lo-L39r-2	AAGAACCATTACTACCTTTTGTATCAG	this study
Lo-L4250	CGGGGTTCAGGAGAGAGTTTAG	this study
Lo-L4251	TTTGCCCTAGAGGTTGCTGTG	this study
Lo-L5100	GAGTCACCGTTTACTATCGCCG	this study
Lo-N4f	TTATGTTAATTTTAGGATGGGGC	this study
Lo-N4f-2	TCTTACTCAAACTGACCTCAAACACC	this study
Lo-N4-N5	ATTTTAGGATGGGGCTATCAG	this study
Lo-N5-CyB	AATAAAATCTGCAGCATCGC	this study
Lo-N5f-3	TGTGTGGATTTCCGTTTATGG	this study
Lo-N5f-4	TTATGATCTAAGTGTGGTCTAGTGTTG	this study
Lo-N5-N4	TAGTTTTAGTATTCTCCTTTGTGGC	this study
Lo-N5N4-2	TAGGGCACTTGTCCATTCTTC	this study
Lo-N5N4-r	CAGAGTAGAAGAATGGACAAGTGC	this study
